# Histone deacetylase inhibitors: A new mode for inhibition of cholesterol metabolism

**DOI:** 10.1186/1471-2164-9-507

**Published:** 2008-10-29

**Authors:** Sridar V Chittur, Niquiche Sangster-Guity, Paulette J McCormick

**Affiliations:** 1 Center for Functional Genomics, University at Albany, State University of New York, Cancer Research Center, One Discovery Drive, Rm 310, Rensselaer, NY 12144, USA; 2 Johns Hopkins University, School of Medicine, 1550 Orleans St, CRBII Rm 456, Baltimore, MD 21231, USA

## Abstract

**Background:**

Eukaryotic gene expression is a complex process involving multiple cis and trans activating molecules to either facilitate or inhibit transcription. In recent years, many studies have focused on the role of acetylation of histone proteins in modulating transcription, whereas deacetylation of these same proteins is associated with inactivation or repression of gene expression. This study explores gene expression in HepG2 and F9 cell lines treated with Trichostatin A (TSA), a potent histone deacetylase inhibitor.

**Results:**

These experiments show that TSA treatment results in clear repression of genes involved in the cholesterol biosynthetic pathway as well as other associated pathways including fatty acid biosynthesis and glycolysis. TSA down regulates 9 of 15 genes in this pathway in the F9 embryonal carcinoma model and 11 of 15 pathway genes in the HepG2 cell line. A time course study on the effect of TSA on gene expression of various enzymes and transcription factors involved in these pathways suggests that down regulation of *Srebf2 *may be the triggering factor for down regulation of the cholesterol biosynthesis pathway.

**Conclusion:**

Our results provide new insights in the effects of histone deacetylases on genes involved in primary metabolism. This observation suggests that TSA, and other related histone deacetylase inhibitors, may be useful as potential therapeutic entities for the control of cholesterol levels in humans.

## Background

Histone deacetylases (HDACs) are important chromatin remodeling enzymes that are generally involved in transcriptional repression [1]. Mammalian HDACs are classified into three main categories depending on their primary homology to *Saccharomyces cerevisiae *HDACs (RPD3, HDA1 and SIR2). Histone deacetylase inhibitors (HDACIs) tend to show equal effects on gene activation and repression [2-4]. HDACIs have been shown to induce differentiation, apoptosis or growth arrest in a variety of transformed cell lines [5]. This is generally attributed to the ability of these inhibitors to induce an open chromatin conformation facilitating transcription of regulatory genes like p21 which inhibit tumor cell growth [6]. These qualities make HDACIs promising targets for chemotherapeutic intervention.

**Figure 1:**
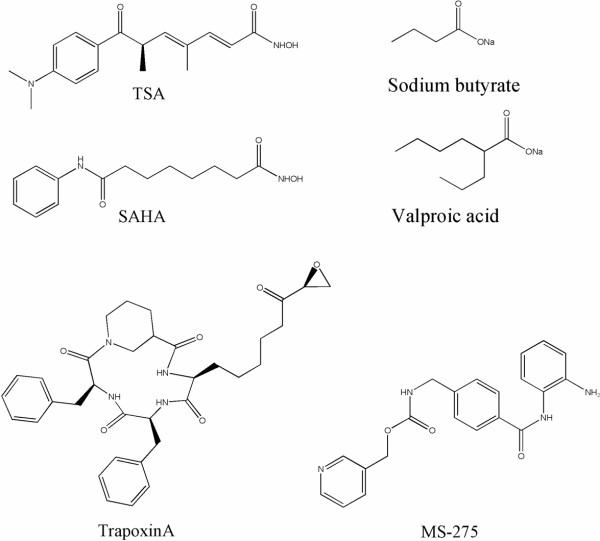
Structures of common HDAC inhibitors.

Recently many different types of HDAC inhibitors have been discovered (Figure 1). These include short chain fatty acids (sodium butyrate, phenylbutyrate, valproic acid) [7], hydroxamic acids (trichostatin A (TSA), suberoylanilide hydromaxic acid (SAHA), pyroxamide, cyclic hydroxamic acid-containing peptides (CHAPs), cinnamic acid bishydroxamic acid (CBHA) and scriptaid) [8,9], cyclic tetrapeptides (trapoxin, apicidin, depsipeptide) [10-13,13], and benzamides (MS-275)[14,15]. Most HDAC inhibitors (HDACIs) developed to date inhibit both Class I and II HDACs equally with the exceptions being valproic acid (5 fold more selective for HDAC1 vs HDACs 5 and 6) and FK-228 (Class I selective). Class I and II HDACs are inhibited by trichostatin A (TSA) and related compounds whereas Class III HDACs are not. As noted, HDACIs have been shown to promote cell cycle arrest, differentiation, and apoptosis in many transformed cultured cell types. In animal models, HDACIs have been shown to inhibit growth of breast, prostate, lung and stomach cancers, as well as neuroblastomas and leukemias, with little toxicity [16,17]. In a previous study looking at the combination regimen of all trans retinoic acid (RA) with the HDACI, Trichostatin A (TSA), we identified several new targets for HDACIs [18]. We also identified critical differences in gene regulation subsequent to treatment with these two agents and a novel promoter module associated with the regulation of a subset of these differentially regulated genes. These analyses focused on the anticancer therapeutic potential of these compounds alone or in combination. Recent analysis of these data identified certain crucial metabolic pathways that have not previously been shown to respond to HDACI treatment and which may be critical in identifying new therapies for cardiovascular health. In this report we discuss the possible role of HDAC inhibition on cholesterol metabolism.

## Results

### Microarray results from F9 cell treatments

**Figure 2:**
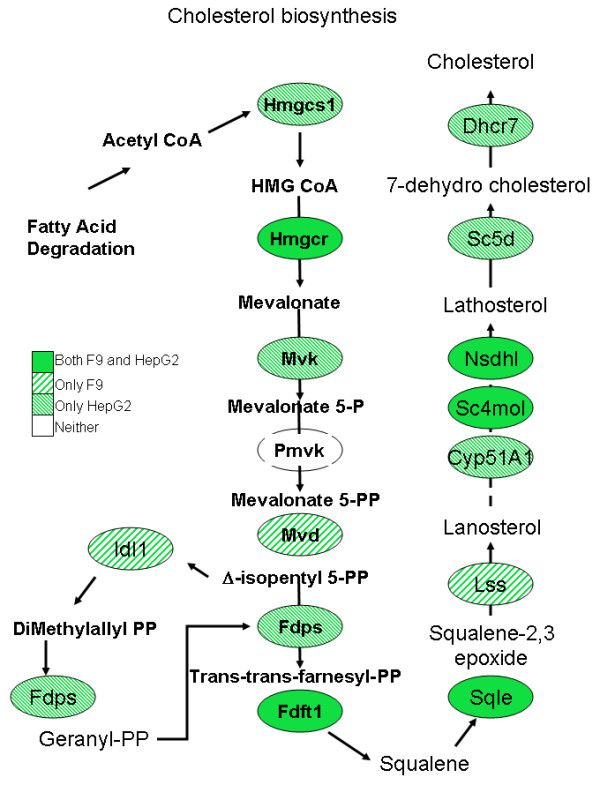
**Map of cholesterol biosynthesis.** TSA down regulates 9 of 15 genes in this pathway in the F9 embryonal carcinoma model and 11 of 15 pathway genes in the HepG2 cell line.

Of the 12,451 mouse genes on the Affymetrix MU74Av2 microarray, 1248 genes (upregulated expression of 489 genes and decreased expression of 759 genes) were found to be significantly differentially expressed following TSA treatment. Of these, only 463 genes were found to be differentially expressed at an arbitrary two-fold or greater level of expression (226 genes up; 237 genes down) (Tables 1 &2, Additional file 1). The raw CEL files for the microarray data are available for download at the Gene Expression Omnibus under series GSE1437. Genes for which up regulated expression was noted were involved in retinoid binding and/or metabolism (e.g., *Crabp2*, *Rbp1*, *Cyp26*), the immune response (*H2-Q7*, *H2-Dma*, *H2-L*, *Cmkor1*, *H-2D4(q)*, *MHC H-2K-f *class 1 antigen); extracellular matrix regulation (*Col5a1*, *Col13a1*, *Gsn*, *Prhp1*, *Tuba3*, *t-PA*, *Cpe*, *Tm4sf6*, *Atp1b2*, *Dsc2*); transcription and maintenance of chromatin structure (*Cbx4*, *Msx2*, *H1f0*, *Elf3*, *Zfpm*), signal transduction (*Il11ra2*, *PLD1*), apoptosis (*Cidea, Zac1*), cell growth regulation (*IGF-II*, *Igfbp3*, *Reck*, *Meis1*, *Scgf*), and embryonic development (*Sema3e*, *Hoxb1 & 4*, *Stra8*, *Hoxa1*, *Cdx-1*). Similarly, genes that were down regulated post-TSA treatment included genes involved in extracellular matrix degradation (*MMP10, Adam23*), transcriptional regulation (*Foxd3*, *UTF1*, *SF1/Nr5a1*, *Msc*, *Mybbp1a*, *HMGI-C*, *lyl1*), signal transduction (*Tdgf1*, *Fst*, *Gna14*, *Il12rb2*, *Il5ra*, *Map3k4*, *Vegfc*), and cell cycle deregulation (*Myb*, *Mybl2*, *Tal1*). Interestingly, we also found down regulated genes involved in pyrimidine biosynthesis (*Dhodh*) and in the cholesterol metabolism pathway (*Mvk*, *Lss*, *Hmgcr*, *Fasn and Sqle*) (Figure 2). This latter finding was intriguing and we decided to extend our investigations beyond the pluripotent mouse EC cells. These experiments were repeated using the more relevant human hepatocarcinoma derived HepG2 cells, since these are hepatic in origin and the liver is the primary source for cholesterol and fatty acid metabolism in humans. While these are not primary hepatocytes, this cell line offers the ability to both explore the hereto unknown effects of TSA on cholesterol metabolism and also look at the previously known targets of this drug.

**Table 1:** Genes upregulated by TSA treatment in F9 cells (representative genes from total of 226 genes at 2-fold level of expression)

**Treatment type**		**EtOH**		**TSA**			
**Systematic**	**FC**	**Norm**	**SE**	**Norm**	**SE**	**Gene**	**GB Acc**

93714_f_at	14.5	1.64	1.0	23.82	3.1	H2-L	AI117211
100127_at	18.0	1.08	0.2	19.37	3.4	CRABP2	M35523
92770_at	18.3	0.83	0.2	15.28	2.0	S100A6	X66449
95471_at	23.2	0.75	0.3	17.30	2.4	CDKN1C	U22399
93981_at	12.8	1.07	0.3	13.72	3.0	PLAT	J03520
92275_at	10.2	1.00	0.3	10.16	0.5	TCFAP2C	X94694
92502_at	7.6	1.00	0.1	7.60	0.8	ZAC1	X95504
100139_at	6.6	0.92	0.3	6.11	0.7	PCSK1N	AI841733
98758_at	6.0	0.82	0.2	4.92	0.4	ALOX15	L34570
160547_s_at	5.7	0.98	0.1	5.60	0.7	TXNIP	AI839138
94545_at	5.5	1.02	0.2	5.60	0.1	RTN1	AW123115
99906_at	4.8	0.93	0.2	4.48	0.4	ESX1	AF085715
93875_at	4.7	1.03	0.2	4.84	0.9	HSP70-3	M12571
161482_f_at	4.7	1.21	0.2	5.65	1.1	PRPH1	AV068234
104716_at	3.8	0.99	0.2	3.82	0.1	RBP1	X60367
99642_i_at	3.6	0.94	0.2	3.40	0.2	CPE	X61232
94881_at	3.3	0.77	0.3	2.59	0.1	CDKN1A	AW048937
99643_f_at	3.2	0.99	0.1	3.16	0.3	CPE	X61232
98067_at	3.2	0.77	0.2	2.43	0.1	CDKN1A	U09507
92501_s_at	3.0	1.01	0.2	3.04	0.5	ZAC1	X95503
93120_f_at	2.9	1.04	0.1	3.01	0.3	H2-K; H-2K	V00746
97487_at	2.8	0.94	0.1	2.60	0.2	SERPINE2	X70296
96704_at	2.6	0.95	0.1	2.52	0.4	SFN	AF058798
93888_at	2.5	0.84	0.2	2.10	0.1	HOXB1; HOX-2.9	X53063
95297_at	2.3	1.06	0.3	2.48	0.3	HOXA1	M22115
93278_at	2.1	1.03	0.2	2.21	0.6	SCP2	M91458
104580_at	2.1	0.79	0.2	1.67	0.1	PLCD	U85711

FC: fold change; Norm: Normalized signal, SE: std error of normalized signal; GB Acc: GeneBank Accession number

**Table 2:** Genes downregulated by TSA treatment in F9 cells (representative genes from total of 237 genes at 2-fold level of expression)

**Treatment type**		**EtOH**		**TSA**			
**Systematic**	**FC**	**Norm**	**SE**	**Norm**	**SE**	**Gene**	**GB Acc**

92889_r_at	-25.8	1.00	0.2	0.04	0.05	FOXD3	AF067421
100700_s_at	-6.3	0.97	0.1	0.15	0.06	NR5A1	AB000490
94712_at	-5.3	1.15	0.2	0.22	0.08	VEGFC	U73620
101578_f_at	-4.9	1.25	0.44	0.26	0.09	ACTB	M12481
99963_at	-4.6	1.26	0.28	0.28	0.11	ZFP101	U07861
93731_at	-4.5	0.96	0.13	0.21	0.07	FKBP9	AF090334
100701_r_at	-3.8	0.94	0.1	0.25	0.07	NR5A1	AB000490
99323_at	-3.7	1.01	0.1	0.27	0.10	IL12RB2	U64199
98817_at	-3.7	1.02	0.2	0.28	0.11	FST	Z29532
99058_at	-3.7	1.05	0.2	0.29	0.11	HMGA2	X99915
95632_f_at	-3.3	0.91	0.3	0.28	0.08	MVK	AW122653
93002_r_at	-3.2	0.86	0.2	0.27	0.07	TDGF1	M87321
102220_at	-2.8	0.90	0.1	0.33	0.08	UTF1; AI505934	AB017360
160737_at	-2.7	1.13	0.2	0.41	0.07	LSS	AW060927
99425_at	-2.6	0.91	0.2	0.35	0.07	HMGCR	X07888
93065_at	-2.6	1.17	0.23	0.45	0.12	IL11RA1	U14412
103683_at	-2.5	0.90	0.2	0.35	0.07	DHODH	AF029667
104285_at	-2.3	1.02	0.2	0.44	0.08	HMGCR	M62766
160832_at	-2.3	0.97	0.16	0.43	0.06	LDLR	Z19521
98575_at	-2.2	1.06	0.2	0.48	0.08	FASN	X13135
94322_at	-2.2	0.97	0.1	0.44	0.07	SQLE	D42048
93234_at	-2.1	1.11	0.2	0.52	0.07	MSC	AF087035

FC: fold change; Norm: Normalized signal, SE: std error of normalized signal; GB Acc: GeneBank Accession number

### Microarray results from HepG2 experiments

**Figure 3:**
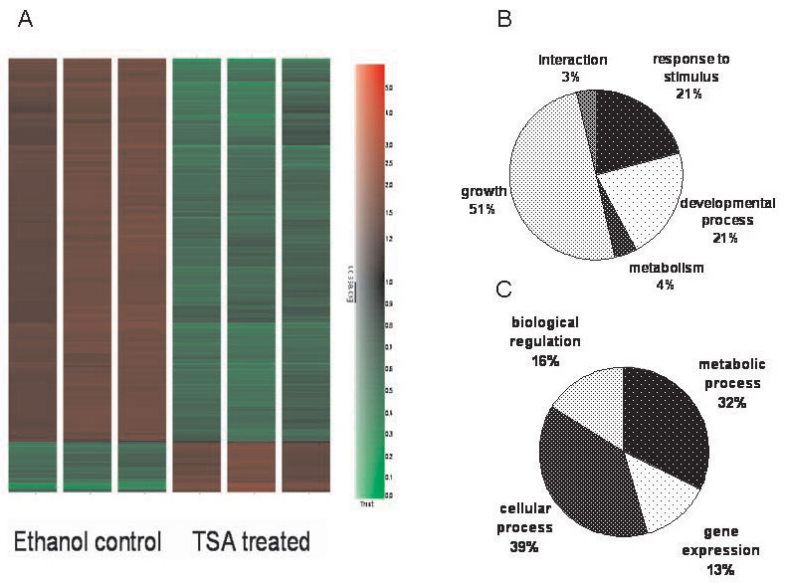
**(A)Hierarchical clustering of HepG2 cells treated with ethanol or TSA shows that the majority of genes are down regulated (green) by TSA treatment in contrast with the current paradigm of the role of HDACs in gene repression.** Gene Ontology analysis of the terms related too "biological process" (p-value < 0.05) shows a significant difference in the genes being up-regulated (B) or down-regulated (C) by TSA. The down regulation of metabolic processes includes cholesterol, lipid and fatty acid metabolism.

Of the 54,613 human genes on the Affymetrix HU133 plus 2.0 array, only 6,513 showed significant differential expression following TSA treatment (p value < 0.05). The raw CEL files for the microarray data are available for download at the Gene Expression Omnibus under series GSE4465. TSA treatment of this cell line resulted in 1561 genes being up regulated and 4952 genes being down regulated at this level of significance (Figure 3). This observation was surprising since the current paradigm of HDAC inhibition suggests equal effects on gene activation and repression after treatment with an HDAC inhibitor [3,4]. Furthermore, using a two-fold cutoff, the genelist was reduced to 3229 genes with 254 up and 2975 down regulated (see Tables 3 and 4; Additional file 1) further emphasizing the extreme extent of gene expression down regulation following HDACI treatment. Genes that showed up regulated expression include phospholipid transfer protein (*Pltp*), tissue inhibitors of metalloproteinase 1 and 2 (*Timp1, Timp2*) and transforming growth factor beta 1 (*Tgfβ1*). Perhaps more importantly, the interesting downregulated genes included thymidylate synthetase (*Tyms*), formyltetrahydrofolate dehydrogenase (*Fthfd*), dihydroorotate dehydrogenase (*Dhodh*) and CTP synthase II (*CTPSII*) (all of which are related to pyrimidine biosynthesis) as well as genes related to lipid transport and fatty acid metabolism including low density lipoprotein receptor (*ldlr*), enoyl-Coenzyme A hydratase, acyl-Coenzyme A dehydrogenase (*Acadm*), apolipoproteins A5, C3, L1, high density lipoprotein binding protein (*vigilin*); and 3-hydroxy-3-methylglutaryl-Coenzyme A synthase 1 (*Hmgcs1*), farnesyl-diphosphate farnesyltransferase 1 (*Fdft1*), squalene epoxidase (*Sqle*), sterol regulatory element binding transcription factor 2 (*Srebf2*) and 7-dehydrocholesterol reductase (*Dhcr7*). Notably 11 of these genes are involved in cholesterol metabolism (Figure 2).

**Table 3:** Genes upregulated by TSA treatment in HEPG2 cells (representative genes from total of 254 genes at 2-fold level of expression)

**Treatment type**		**EtOH**		**TSA**			
**Systematic**	**FC**	**Norm**	**SE**	**Norm**	**SE**	**Gene**	**GB Acc**

214023_x_at	18.5	1.00	0.1	18.61	1.2	TUBB	AL533838
201008_s_at	10.2	0.94	0.1	9.60	1.1	TXNIP	AA812232
227404_s_at	9.5	1.00	0.1	9.50	0.3	EGR1	AI459194
218280_x_at	6.5	0.92	0.1	5.97	0.4	HIST2H2AA	NM_003516
221059_s_at	4.6	0.95	0.1	4.36	0.2	CHST6	NM_021615
208581_x_at	4.4	0.96	0.1	4.20	0.2	MT1X	NM_005952
203158_s_at	3.9	1.00	0.1	3.91	0.2	GLS	AF097493
206907_at	3.7	1.09	0.1	4.07	0.3	TNFSF9	NM_003811
202075_s_at	3.6	0.98	0.1	3.48	0.2	PLTP	NM_006227
201666_at	2.8	1.01	0.1	2.84	0.2	TIMP1	NM_003254
203085_s_at	2.3	1.02	0.1	2.35	0.2	TGFB1	BC000125

203167_at	2.3	1.00	0.1	2.25	0.0	TIMP2	NM_003255

FC: fold change; Norm: Normalized signal, SE: std error of normalized signal; GB Acc: GeneBank Accession number

**Table 4:** Genes downregulated by TSA treatment in HEPG2 cells (representative genes from total of 2975 genes at 2-fold level of expression)

**Treatment type**		**EtOH**		**TSA**			
**Systematic**	**FC**	**Norm**	**SE**	**Norm**	**SE**	**Gene**	**GB Acc**

205890_s_at	-30.7	0.975	0.05	0.032	0.02	UBD	NM_006398
220437_at	-12.6	0.976	0.08	0.078	0.06	LOC55908	NM_018687
223493_at	-11.5	1.056	0.07	0.092	0.06	FBXO4	AF129534
226388_at	-11.4	1.015	0.06	0.089	0.03	TCEA3	AI675780
202589_at	-9.6	1.07	0.09	0.11	0.04	TYMS	NM_001071
203979_at	-8.7	1.007	0.08	0.115	0.05	CYP27A1	NM_000784
226216_at	-7.2	0.887	0.11	0.123	0.06	INSR	W84556
209608_s_at	-6.3	1.034	0.06	0.163	0.03	ACAT2	BC000408
203924_at	-6.2	0.974	0.05	0.158	0.04	GSTA2	NM_000846
205208_at	-6.9	1.03	0.08	0.15	0.06	FTHFD	NM_012190
219366_at	-5.6	0.954	0.14	0.169	0.06	AVEN	NM_020371
205820_s_at	-5.3	1.02	0.06	0.19	0.04	APOC3	NM_000040
209546_s_at	-5.2	0.99	0.06	0.19	0.07	APOL1	AF323540
224243_at	-4.6	1.10	0.12	0.24	0.05	APOA5; RAP3	AF202889
200789_at	-4.0	0.96	0.11	0.24	0.03	ECH1; HPXEL	NM_001398
221750_at	-3.8	0.99	0.05	0.26	0.04	HMGCS1	BG035985
202068_s_at	-3.6	1.00	0.06	0.28	0.04	LDLR; FH; FHC	NM_000527
225012_at	-3.1	0.99	0.09	0.32	0.04	HDLBP	BE378479
213577_at	-3.1	1.03	0.09	0.34	0.05	SQLE	AA639705
202067_s_at	-2.9	0.88	0.12	0.30	0.16	LDLR	AI861942
202502_at	-2.8	0.97	0.05	0.35	0.03	ACADM; MCAD;	NM_000016
201248_s_at	-2.6	1.00	0.08	0.39	0.04	SREBF2; SREBP2	NM_004599
209218_at	-2.5	1.01	0.06	0.40	0.04	SQLE	AF098865
222916_s_at	-2.3	0.92	0.12	0.40	0.06	HDLBP	AF116718

201791_s_at	-2.2	0.99	0.07	0.45	0.04	DHCR7; SLOS	NM_001360

FC: fold change; Norm: Normalized signal, SE: std error of normalized signal; GB Acc: GeneBank Accession number

### Quantitative PCR results

**Figure 4:**
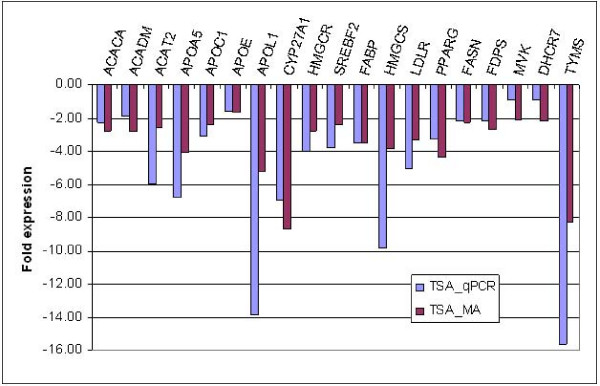
**Real Time qPCR verification of the gene expression levels of various genes involved in (A) lipid transport and fatty acid synthesis, (B) cholesterol metabolism and (C) pyrimidine biosynthesis in HepG2 cells.** Fold expression is relative to ethanol control. TSA treatment showed down regulated expression of 14 genes by both qPCR and microarray.

**Figure 5:**
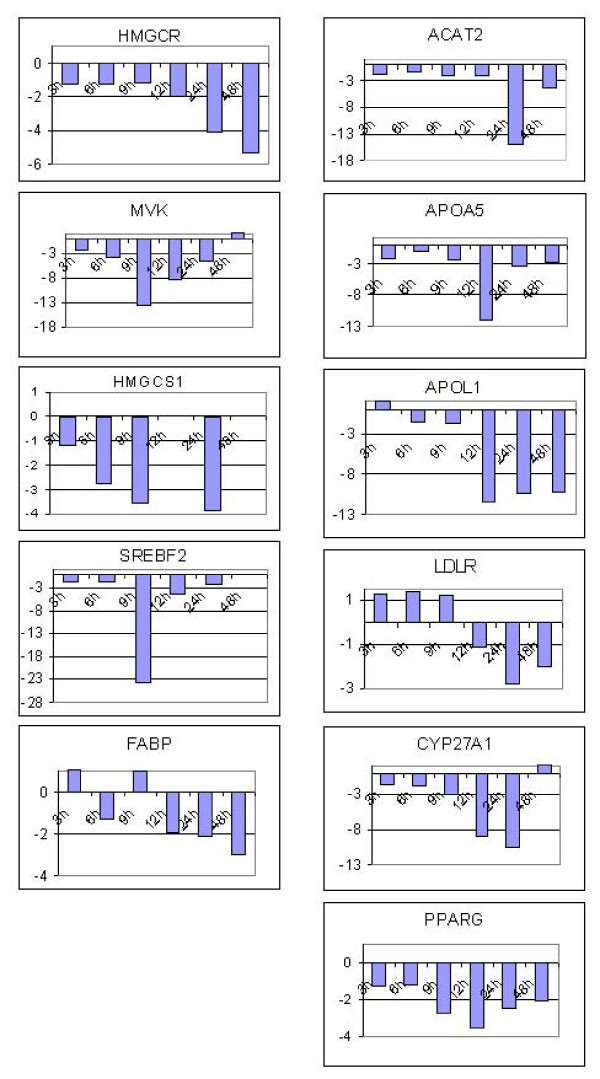
**Time course of gene expression in response to TSA treatment of HepG2 cells for 3, 6, 9, 12, 24 and 48 h. **Values are represented as fold change relative to ethanol treated controls at the respective time points.

Sybr green qPCR was used to validate microarray expression data for a subset of the differentially expressed genes. The expression patterns of 10 genes from the F9 microarray data set and 21 from the HepG2 microarray data set (Figure 4) were all confirmed by qPCR. Furthermore we decided to examine the levels of gene expression at early and late time points for 11 of these genes that have a role in cholesterol and lipid metabolism. The relative gene expression was obtained for these genes at 3 h, 6 h, 9 h, 12 h, and 48 h to serve as early and late time frames in comparison to the 24 h treatments (Figure 5). *Hmgc*r which is the rate limiting enzyme in cholesterol biosynthesis was repressed 2-fold after 12 h of TSA treatment and showed increasing down regulation over 24 h (4 fold) and 48 h (5.3 fold) time points. *Hmgcs *levels showed increased repression (2.8–3.8 fold) by TSA treatment over 6–24 hours (the amplification reactions failed for the 12 h and 48 h time points). Levels of *Mvk *(2.1 fold) and *Srebf2 *(1.8 fold) were down regulated at 3 h with maximal repression at 9 h (13.6 fold and 23.6 fold respectively) after which the levels then came back to normal over the next 39 hours. *Srebf2 *levels at 6 h, 12 h and 24 h were 2.4 fold, 4.5 fold and 2.4 fold respectively. Genes involved in lipid and fatty acid metabolism such as *ApoA5 *and *Acat2 *were found to be maximally down regulated at 12 h (12 fold) and 24 h (16 fold) time points respectively while *ApoL1 *was down regulated (11 fold) at 12, 24 and 48 h time points. *Fabp *which is involved in fatty acid metabolism showed increasing down regulation after 12 h (1.9 fold) while *Pparγ *was found to be increasingly repressed at 9 h (2.8 fold) followed by reversal after 12 h (3.5 fold). The *Pparγ *levels after 48 h of TSA treatment were still almost 2 fold down regulated as compared to untreated cells. Levels of *Cyp27A1 *or sterol 27-hydroxylase which participates in the conversion of cholesterol to bile acids was also found to be initially down regulated at 6 h (1.8 fold) and increasingly over the 12 (8.8 fold) and 24 h (10.3 fold) time points. TSA treatment did not show any significant effect on *Ldlr *expression until 24 h (2.8 fold).

## Discussion

In a previous study we had used microarray analyses to examine the effects of RA and TSA on embryonal carcinoma cell growth and differentiation using the prototypical EC cell line F9 [18]. Results from these studies identified several important genes and pathways differentially regulated by these compounds. In this report we identify new target pathways for TSA treatment based on further analysis of this data. Most importantly, the regulatory pathways that are affected include pyrimidine metabolism and cholesterol biosynthesis. The pyrimidine pathway is of interest because one of the rate limiting enzymes in this pathway, dihydroorotate dehydrogenase (*dhodh*), has been targeted for inhibition in murine models of rheumatoid arthritis as well as in the human T-lymphoblastoma cell line (A3.01) [21,22]. *Dhodh *catalyzes the fourth committed step in the de novo biosynthesis of pyrimidines. Activated lymphocytes expand their pyrimidine pool by eightfold during proliferation [23]. In rheumatoid arthritis, inflammation and degradation of synovial tissue are initiated by the influx of lymphocytes (B cells, CD4+, CD8+ and T cells) [24]. Thus, inhibiting activated T-cells by decreasing their supply of pyrimidines via TSA treatment could provide an attractive alternative method for treating rheumatoid arthritis. Interestingly, HDACIs (TSA and phenylbutyrate) were used as treatments in a rat model of rheumatoid arthritis, and resulted in reduced inflammation, and inhibition both of synovial hyperplasia and bone or cartilage destruction [25]. The authors also found that HDACIs inhibited the expression of tumor necrosis factor-α, which functions to stimulate matrix degradation in rheumatoid arthritis [26], therefore suggesting a mechanism by which HDACIs may alleviate some effects of rheumatoid arthritis. Further extending and supporting these results, in this study, we found that TSA itself could significantly inhibit the expression of *dhodh *even in non-lymphatic cells (60% in F9 and 25% in HepG2), providing an alternative (or synergistic) mechanism by which HDACI might suppress rheumatoid arthritis in both mice and men. Moreover, the mRNA levels of thymidylate synthetase (*Tyms*), another key enzyme in this pathway, were decreased 8 fold in HepG2 cells (2.4 fold in F9 cells) further potentiating this effect of TSA treatment. A previous study using chondrocytes showed that HDACIs such as TSA and sodium butyrate, blocked the induction of matrix metalloproteinases (*MMP-1, MMP-13*) as well as aggrecan-degrading enzymes (*Adamts4, Adamts5 and Adamts9*) [27]. Both of these enzyme families mediate cartilage destruction. In our study with HepG2 cells, we also found that TSA treatment resulted in a modest decrease in the expression of MMPs (*MMP-1, MMP-2, MMP-11, MMP-12*) (1.2–1.5 fold) and *Adamts9 *(1.6 fold). This, coupled with increased expression (2.8 fold) of a collagenase inhibitor (tissue inhibitor of MMP: *TIMP1, TIMP2*), might further promote maintenance of a growth regulating matrix. Interestingly, TSA treatment also resulted in down regulation of LPS induced *TNFα *levels (2 fold) as well as suppression of the cytokines *IL-12 *and *IL-8 *(2.7 and 2 fold respectively). This result is consistent with a report by Leoni *et al*. [28] on the anti-inflammatory properties of SAHA (another hydroxamic acid based HDACI) in Balb/c mice and human PBMCs induced with LPS.

The pathway most significantly affected by TSA treatment in F9 EC cells is that of cholesterol biosynthesis, most specifically those steps involved in the synthesis of low density lipoprotein. There are two main types of lipoproteins that transport cholesterol in the blood: low density lipoproteins (LDL) and high density lipoproteins (HDL). HDL particles are generally considered to be "good cholesterol", while LDL is considered "bad cholesterol" [29]. Several genes encoding essential enzymes in the LDL synthesis pathway are down regulated by these treatments. Pathway analysis of microarray data using genes showing statistically significant (p < 0.05) differential gene expression indicated that expression levels of 9 enzymes out of the 15 in the cholesterol biosynthesis pathway are decreased following TSA treatment. They include HMG CoA reductase (*Hmgcr*), mevalonate kinase (*Mvk*), di-p-mevalonate decarboxylase (*Mvd*), isopentenyl-PP isomerase (*Idi1*), squalene synthatase (*Fdft1*), squalene epoxidase (*Sqle*), lanosterol synthase (*Ls*s) and lanosterol oxidase (*Sc4mol*) and NAD(P)-dependent steroid dehydrogenase (*Nsdhl*) (Figure 2). Of these only 6 of these 9, including *Hmgcr*, *Mvd*, *Idi1*, *Sqle*, *Lss*, *Sc4mol *and *Nsdhl*, showed a greater than two fold differential expression on TSA treatment. The *z*-score assigned to each category by MAPPFinder reflects the degree to which the expression of genes in that category was greater than that expected by chance. A positive *z*-score indicates that a large number of genes in that category are differentially expressed between the compared conditions while a negative Z score indicates that the there are fewer genes meeting the criterion than would be expected by random chance. If the MAPPFinder data truly obeyed the assumptions of the hypergeometric distribution, then a Z score or 1.96 or -1.96 would correlate with a p value of 0.05. The z-score for this pathway was highly significant with values of 4.85 associated with TSA treatment. Real-time qPCR analyses verified decreased expression of 3 of 5 genes (*Hmgcr, Mvd, Lss*) in this pathway but the expression of neither *Mvd *nor *Lss *was significantly down regulated by TSA. Decreasing high cholesterol levels using TSA treatments may work well since repression of few of the detected genes may be sufficient to induce the response, i.e., a reduction in cholesterol intermediates and synthesis.

Following the analysis of these pluripotent EC cells, we decided to investigate these effects in the HepG2 cell line which arose from a carcinoma of the human liver, the primary organ for cholesterol and fatty acid metabolic processes. While we realize that primary hepatocytes would be a better model to evaluate this pathway, we choose the HepG2 cell line as an means to evaluate this phenomenon but allowing for use of the known anti-cancer effects of TSA as a control. Expression data from HepG2 cells also indicated that multiple enzymes in cholesterol biosynthesis and fatty acid synthesis pathways were significantly down regulated (Figure 3). The mRNA transcript levels that were repressed at a greater than 2 fold level of significance included HMG CoA synthase (*Hmgcs1*), HMG CoA reductase (*Hmgcr*), sterol receptor binding factor-2 (*Srebf2*) and lanosterol 14 α-demethylase (*Cyp51a1*) (involved in cholesterol metabolism), and others including fatty acid synthase (*Fasn*), fatty acid binding protein (*Fabp*), farnesyl diphosphate synthase (*Fdps*), acetyl-coA carboxylase (*Acaca*), acetyl-coA dehydrogenase (*Acadm*), acetyl-coA acetyl transferase (*Acat2*), peroxisome proliferative activated receptor, gamma (*Pparγ*) and a variety of apolipoproteins that are involved in fatty acid and triglyceride metabolism. Quantitative PCR studies verified that TSA treatment reduced expression of *Hmgcr, Hmgcs1, Srebf2, Fabp Fasn, Fdps, Acaca, Acadm, Acat2, ApoA5, C1, E *and *L1 *as well as *Cyp27a1*, *Ldlr*, *Pparγ and Tyms *(Figure 4). The down regulation seems to be a complex phenomenon involving genes that regulate these pathways at different levels. Most evident is the down regulation of *Srebf2 *which in turn acts as a transcription factor regulating the expression of enzymes like *Hmgcr *(the target for the statin class of drugs) and *Mvd*. It is known that *Srebf2 *overexpression induces all 12 enzymes in the cholesterol biosynthesis pathway and inhibition of *Srebf2 *by TSA might inhibit the expression of these enzymes [30]. In fact, our microarray data demonstrates that the levels of almost all these enzymes are down (10 genes pass all cutoff filters) following TSA treatment. The repression of *Srebf2 *occurs at a early time point (around 3 hrs) and continually repressed over 24 hrs (2.4 fold repression at 24 h). This effect is probably responsible for the down regulation of the cholesterol pathway since expression of *Hmgcs *(repressed at 6 h), *Hmgcr *(repressed at 12 h) and *Ldlr *(repressed at 24 h) are all known to be induced by Srebf2 [31]. *Srebf-1a *and -*1c *are more involved with regulation of fatty acid synthesis and lipogenesis [30,32]. While we were unable to detect the levels of *Srebf1 *expression in our microarray experiments, TSA treatment modestly (1.3 fold) decreased expression levels of cytosolic NADP-dependent isocitrate dehydrogenase1 (*Idh1*) which provides the cytosolic NADPH required for proper functioning of both the cholesterol and fatty acid biosynthetic pathways. The *Idh1 *promoter is activated by *Srebf1a *and *Srebf2 *in human hepatoma cells [33]. Thus there appears to be a concerted down regulation of both pathways through a synergistic effect. *Cyp51a1 *(lanosterol 14α-demethylase) is another important intermediate in cholesterol metabolism and has in recent years gained importance as a target for the development of hypocholesterolemic agents [34]. Our microarray data showed that levels of *Cyp51a1 *were also down regulated further adding support to the possible use of this HDACI as a way to decrease plasma cholesterol levels.

Finally, atherosclerosis is the underlying disorder associated with most cardiovascular disease [35]. This disorder is characterized by deposits of fatty substances, cholesterol, cellular waste products, calcium and other substances in the inner lining of an artery (collectively known as plaques) [36]. Cholesterol has been implicated as the major contributor to this condition as atherosclerosis is strongly correlated with an increase in serum cholesterol levels [37,38]. Generally, serum levels should be between 140 and 200 mg per deciliter (mg/dl) whereas high levels surpassing 240 mg/dl indicate one is at high risk for cardiovascular disease [39]. Thus, atherosclerosis is characterized by elevated levels of LDL [40]. The activity of the hepatic LDL receptor (Ldlr) is the primary determinant of plasma LDL cholesterol levels and *Ldlr *transcription is in turn regulated by *Srebf2*. When the levels of hepatocellular sterols drop, *Srebf2 *is activated and this process restores the normal levels by concurrent activation of *de novo *cholesterol synthesis and increased uptake of plasma cholesterol through Ldlr. LDL receptor is also post transcriptionally regulated by proprotein convertase subtilisn/kexin type 9a (*Pcsk9*) in an inverse manner [41]. While our microarray and qPCR data shows decreased *Ldlr *expression following TSA treatment, microarray gene expression levels of *Pcsk9 *are also down regulated. This suggests existence of a mechanism for potential compensatory increase in Ldlr levels or activity post-transcriptionally. Our time course experiments did not show a significant repression of *Ldlr *levels until 24 h further highlighting the complex nature of *Srebf2 *regulation.

Our data with TSA treatment also showed a decrease in the levels of gene expression for a variety of apolipoproteins including *apoA1, apoA5, apoB, apoC1, apoE, apoL1*. This observation highlights the complex relationship of apolipoprotein levels and lipoprotein metabolism. While elevated levels of apoB and reduced levels of apoA1 are associated with increased cardiac disease, serum levels of apoB100 associated VLDL are regulated in turn by Acat2 which stimulates cholesteryl ester secretion into apoB-containing lipoproteins. Acat inhibitors are being developed as a therapeutic means to lower LDL cholesterol without affecting cholesterol uptake [42,43]. Also apoE deficient mice show high levels of cholesterol and develop spontaneous atherosclerosis while mice with partial or complete deficiency of high-mobility group A2 protein (Hmga2) are able to resist diet-induced obesity [44]. *Acat2 *inhibition using antisense nucleotides was previously shown to alleviate atherosclerosis in *apoB-Ldlr *-/- mice [45]. This study also found Acat2 inhibition to be effective in reducing plasma cholesterol, increasing plasma triglycerides, and shifting LDL cholesteryl ester fatty acids to become mainly polyunsaturated. In our study, TSA treatment showed a modest decrease in *apoB, apoE *and *apoA1 *in addition to decreased levels of *Acat2, Fasn and Hmga2*. This indicates that triglyceride metabolism is perturbed by TSA and further studies may be necessary to evaluate the possibility of using TSA and other HDACIs for modulating triglyceride metabolism. *Cyp2*7 has been reported to be regulated by the nuclear receptor subfamily of which *PPARγ *is a member and levels of both these genes have been found to be high in atherosclerotic lesions. Levels of *Cyp27a1 *(maximal repression at 24 h) and *Pparγ *(maximal repression between 9–12 h) were found to be repressed by TSA treatment in both the microarray and qPCR data. This observation adds credence to the potential for development of TSA like HDACIs for atherosclerosis.

## Conclusion

Our results show that cholesterol metabolism is significantly down regulated by TSA both directly and indirectly and thus HDACI therapy may be a relatively novel tool to develop for use in controlling cholesterol levels. This study only addresses the effect of TSA treatment on transcript levels of the rate limiting enzymes and transcription factors and further studies evaluating protein expression levels are necessary to derive firm conclusions on regulation of this pathway. Additional studies exploring the different classes of HDACIs with respect to their effects on regulation of the genes in the cholesterol pathway would also help dissect the details of this innovative application for these drugs.

## Methods

### Cell Culture for Microarray and Quantitative PCR Analysis

F9 mouse embryonal carcinoma cells were cultured as published previously [18] Stock solutions of TSA (3 mM) (Sigma-Aldrich) were freshly prepared in absolute ethanol for each experiment and were diluted in DMEM to a final concentration of 70 nM. Cells were seeded at 2.5 × 10^6 ^cells/75 cm^2 ^gelatinized flask and treated with ethanol or TSA for 24 h. All experiments were performed in triplicate using a different preparation of F9 cells for each experiment.

Similarly, HepG2 human hepatoma cells were cultured using DMEM containing 10% FBS and treated with TSA (0.35 μM) or an ethanol control (final concentration 0.2%) for 24 hours before being harvested for RNA isolation. For time course experiments, total RNA was isolated from HepG2 cells treated with an ethanol control or 0.35 μM TSA for 3, 6, 9, 12, 24 or 48 h.

### RNA extraction and purification

Both F9 and HepG2 cells were harvested with 4 mL of Tri-reagent (Molecular Research Center, Inc) and RNA isolation was carried out according to the manufacturer's protocol. Total RNA was purified using the RNeasy cleanup kit and protocol (Qiagen), quantified and then analyzed for degradation on a BioAnalyzer (Agilent).

### Hybridization of sample to GeneChip Microarrays

RNA was converted to biotinylated cRNA (complimentary RNA) from oligo-dT-primed cDNA using standard Affymetrix protocols. Biotinylated cRNA was used to probe the MU74Av2 (F9 samples) or HU133 Plus 2.0 (HepG2 samples) Affymetrix GeneChip microarrays. A total of six samples (three controls and three TSA treated) for each cell line were analyzed.

### Statistical Analysis

The raw data (CEL files) were imported into GeneSpring software (v7.2) for further analysis. A two-step normalization algorithm was implemented to select differential gene expression in response to TSA samples (ethanol treated samples as baseline). In the first normalization step, a global scaling per chip method was used in which the signal of each gene was divided by the mean intensity (50^th ^percentile) of the chip. This normalization step was followed by a per gene normalization which divides each gene by the average intensity of that gene in several control samples. Hierarchical clustering was used to organize the data in discrete expression profiles. Selection of statistically significant genes from each expression profile was done using a p-value cut off of ≤ 0.05 with the cross gene error model (CGEM) combined with Welch t-test. The multiple testing correction (Benjamini and Hochberg false discovery rate) was integrated within each test. Additionally we also analyzed the HepG2 data using both MAS5 (Microarray Suite) as well as RMA (Robust Microarray Analysis) algorithms and genes that passed all criteria from both sets of analyses were used for follow up studies.

### Pathway and Functional Cluster Analysis

The differentially expressed genes selected as described above were subjected to functional cluster analysis using MAPPFinder in conjunction with GenMAPP (Gene Microarray Pathway Profiler) 2.0 [19,20]

### Quantitative Real-Time PCR

To verify the data obtained from microarrays, 5 μg of total RNA was taken from the same pool of RNA as used for the microarray experiments. The RNA was DNase treated (Ambion) and reverse transcribed to cDNA which served as the template for quantitative PCR (qPCR). Real-time relative qPCR (SYBR Green; Applied Biosystems) was performed in triplicate using a GeneAmp 5700 (F9 samples) or a HT7900 sequence detection system (HepG2 samples) according to the manufacturer's instructions. Primers were specifically designed using Primer express software (Applied Biosystems). 1 μg of cDNA was amplified in 1× SYBR green buffer. PCR conditions were: 10 min at 95°C for AmpliTaq Gold DNA polymerase activation, 45 thermal cycles of 15 sec at 95°C to denature and 1 min at 60°C to anneal and extend. Relative expression levels were analyzed using the $2^{-\Delta \Delta {\text{C}}_{\text{T}}}$ method using the GAPDH expression level as a control. Samples treated with TSA were compared to the baseline expression value determined from ethanol treated samples at the respective time points and the fold change is shown. (For primer sequences see Table 5).

**Table 5:** Primers used for Sybr green qPCR

**F9 cells**		
**Gene**	**Forward**	**Reverse**
GAPDH	5'-GCCAAGAGGGTCATCATCTCC-3'	5'-TTGGTTCACACCCATCACAAA-3'
MVD	5'-AGCATCGCCCGGCAG-3'	5'-TGGCCCCTGTAATTTCCCA-3'
LSS	5'-GCGGCTGTGCGATGCT-3'	5'-AGGTAGCGAACCCGCCA-3'
TGFB1	5'-TGGAAAGGGCCCAGCAC-3'	5'-GCAATAGTTGGTATCCAGGGCT-3'
IGFII	5'-AAGAGTTCAGAGAGGCCAAACG-3'	5'-ATCTCCGAAGAGGCTCCCC-3'
MT1	5'-TGCTCCACCGGCGG-3'	5'-TTTGCAGACACAGCCCTGG-3'
WNT6	5'-GGGCGCTGTCTGAGTCCA-3'	5'-TGGCCCCTGTAATTTCCCA-3'
tPA	5'-GGCCTGGCACGACACAAT-3'	5'-CATCACATGGCACCAAGGTC-3'
VEGFC	5'-CAGCTGCGGAAAGGCG-3'	5'-TTTACACTGTCCCCTGTCCTGG-3'
DHODH	5'-AACACAGGCTACGGGCCAG-3'	5'-TCCCAGAGGCAGGCCCAT-3'
		
**HepG2 cells**		
**Gene**	**Forward**	**Reverse**
ACADM	5'-AGCTACCAAGTATGCCCTGGAA-3'	5'-TAAATGATATTGCTTGGTGCTCTACA-3'
ACAT2	5'-TGGGCCACCCTCTTGGA-3'	5'-CCAGTGTGTGTAACAGGGTCACA-3'
ACACA	5'-GCTCCTTGTCACCTGCTTCTG-3'	5'-TGTAGGCTAGAGATCCCCAAATCA-3'
APOA5	5'-AGGTGCGCCAGCGACTT-3'	5'-GCGAGTGAAGGCAGCTATCTG-3'
APOC1	5'-CAAGGCTCGGGAACTCATCA-3'	5'-CCCGCATCTTGGCAGAAA-3'
APOE	5'-CGCTGGGTGCAGACACTGT-3'	5'-AGGCCTTCAACTCCTTCATGGT-3'
APOL1	5'-TCAGCTGAAAGCGGTGAACA-3'	5'-CTCTGCTCATTTCCAGGATGCT-3'
CYP27A1	5'-CCCTGTGGTCCCCACAAA-3'	5'-GGAAGCCATCAACTTCAATTTCC-3'
HMGCR	5'-CCTGTAACTCAGAGGGTCAAGATGAT-3'	5'-CCAGCGACTGTGAGCATGAA-3'
HMGCS	5'-TCTTAAATCAAGGCTTGATTCAAGAA-3'	5'-TGTCCTCTCTGAGCTTCATGTTTT-3'
SREBF2	5'-CGAATTGAAAGACCTGGTCATG-3'	5'-TCCTCAGAACGCCAGACTTGT-3'
FABP	5'-CCGCTGGGTCCAAAGTGAT-3'	5'-CATTGTCTCCAGCTCACATTCC-3'
FASN	5'-GCAAATTCGACCTTTCTCAGAAC-3'	5'-GGACCCCGTGGAATGTCA-3'
LDLR	5'-AGATAGTGACAATGTCTCACCAAGCT-3'	5'-CTCACGCTACTGGGCTTCTTCT-3'
PPARG	5'-GCGAAAGCCTTTTGGTGACT-3'	5'-CAGTGCATTGAACTTCACAGCAA-3'

TYMS	5'-AATCACATCGAGCCACTGAAAA-3'	5'-AATCCTGAGCTTTGGGAAAGGGT-3'

For quantitative analysis of the data, C_T _(threshold-cycle number) values were normalized to those of *GAPDH*, with use of the ΔΔC_T _method.

## Authors' contributions

SVC conceived the study design for the HepG2 samples and conducted all the analysis for this paper. NSG performed the microarray study on the F9 samples under the guidance of PJM.

## Supplementary Material

Additional file 1 **Full list of statistically significant (p < 0.05) genes differentially expressed (2-fold or greater) in F9 and HepG2 cells on TSA treatment.** Click here for file
